# Have Therapeutics Enhanced Our Knowledge of Axial Spondyloarthritis?

**DOI:** 10.1007/s11926-023-01097-7

**Published:** 2023-01-18

**Authors:** S. R. Harrison, H. Marzo-Ortega

**Affiliations:** 1 The University of Leeds, Leeds Institute for Rheumatic and Musculoskeletal Medicine (LIRMM), NIHR Leeds Biomedical Research Centre, Leeds Teaching Hospitals Trust, Leeds, UK; 2 The University of Leeds, Leeds Institute of Cardiovascular and Metabolic Medicine, the LIGHT building, Clarendon Way, Leeds, UK

**Keywords:** Axial spondyloarthritis, Ankylosing spondylitis, Biological disease-modifying antirheumatic drugs (bDMARDs), Therapeutics

## Abstract

**Purpose of Review:**

An overview of how the treatment landscape of axial spondyloarthritis (axSpA) has shaped our understanding of the disease.

**Recent Findings:**

Prior to the millennium, non-steroidal anti-inflammatory drugs (NSAIDs) were the only treatment for axSpA, yet only 30% of patients responded and many developed side effects. In 2003, the first biological disease-modifying drug (bDMARD) was licensed for axSpA which substantially improved outcomes in comparison to NSAIDs. In 2022, there are now several bDMARDs for axSpA; however, they too are not universally efficacious in treating axial inflammation and may have deleterious effects on extramusculoskeletal manifestations. Nevertheless, successful or not, each bDMARD gives invaluable insight into axSpA immunobiology.

**Summary:**

This review discusses how much we have learned from the use of bDMARDs in axSpA, how this has redefined our understanding of the disease, and how we might use this knowledge to develop new and better treatments for axSpA in the future.

## Introduction

Axial spondyloarthritis [axSpA; formerly ankylosing spondylitis(AS)] is an inflammatory arthritis affecting primarily the sacroiliac joints (SIJs) and spine, and is considered to be the prototype of a group of clinically and genetically related diseases called the seronegative spondyloarthropathies (SpA). The wider SpA family now includes radiographic-axSpA (r-axSpA/AS), non-radiographic(nr)-axSpA, psoriatic arthritis (PsA), reactive arthritis and enteropathic arthritis [1]. Clinical features include inflammatory back pain with or without peripheral manifestations (arthritis, enthesitis and dactylitis), and extra-musculoskeletal manifestations [uveitis, psoriasis and inflammatory bowel disease (IBD)]. Historically, the diagnosis depended on the presence of characteristic bone changes of the sacroiliac joints and/or spine on plain film X-rays [2]; however, in recent decades MRI has enabled the detection of spinal inflammation without bony disease. Accordingly, in 2011, The Ankylosing Spondylitis Association (ASAS) published new classification criteria distinguishing between those with prototypical plain film changes (r-axSpA) and those with only clinical and/or MRI features of the disease (nr-axSpA) [3].

The first-line treatment for axSpA is non-steroidal anti-inflammatory drugs (NSAIDs) or cyclooxygenase 2(COX-2) inhibitors [4]; however, approximately a third of patients fail to respond or are intolerant to these agents [5, 6]. In spite of this, NSAIDs/ COX-2 inhibitors remained the only pharmacological options for patients for decades, until 2003, when the first biological disease-modifying anti-rheumatic drug (bDMARD), the TNFα inhibitor (TNFi) etanercept, received marketing authorisation for r-axSpA [7]. Since then, several biologics have been added to the therapeutic arsenal, which now includes five TNFis (etanercept, infliximab, adalimumab, golimumab, certolizumab and their biosimilars), two IL-17A inhibitors (secukinumab and ixekizumab), and two Janus Kinase inhibitors (JAKi) (tofacitinib and upadacitinib) [8–13]. Other agents in development and/or close to receiving market authorisation include brodalumab (IL-17 receptor blocker) [14], bimekizumab (IL-17A + IL-17F inhibitor) [15–18], namilumab ([granulocyte-macrophage colony-stimulating factor (GM-CSF)] inhibitor) [19] and filgotinib (JAKi) [20]. Contrastingly, IL-23 inhibitors were unsuccessful in Phase III trials, despite the positive result in early open-label studies [21–24].

Before reading this review, it is useful for the reader to appreciate some important caveats about drug development and discovery in axSpA. Most early bDMARDs and their molecular targets were conceptualised in other immune-mediated inflammatory diseases (IMIDs) and extrapolated to axSpA. The TNFis were first trialled in axSpA on the premise that axSpA might share common inflammatory pathways with rheumatoid arthritis (RA) given the emerging evidence of inflammatory lesions on the SIJ [25]. Molecular studies then followed identifying other cytokines including IL-1 and IL-6 in the serum of axSpA patients [26–28]. Yet, evidence for IL-1 inhibition in axSpA is limited with only two small open-label studies performed in ankylosing spondylitis (r-axSpA) in the mid-2000s [29, 30]. Later, Phase II/III trials of tocilizumab and sarilumab (IL-6is) failed to reach their endpoints [31, 32], despite real-world evidence suggesting that IL-6i may have a role in a subset of synovial-driven refractory spondyloarthritis [33].

**Fig. 1:**
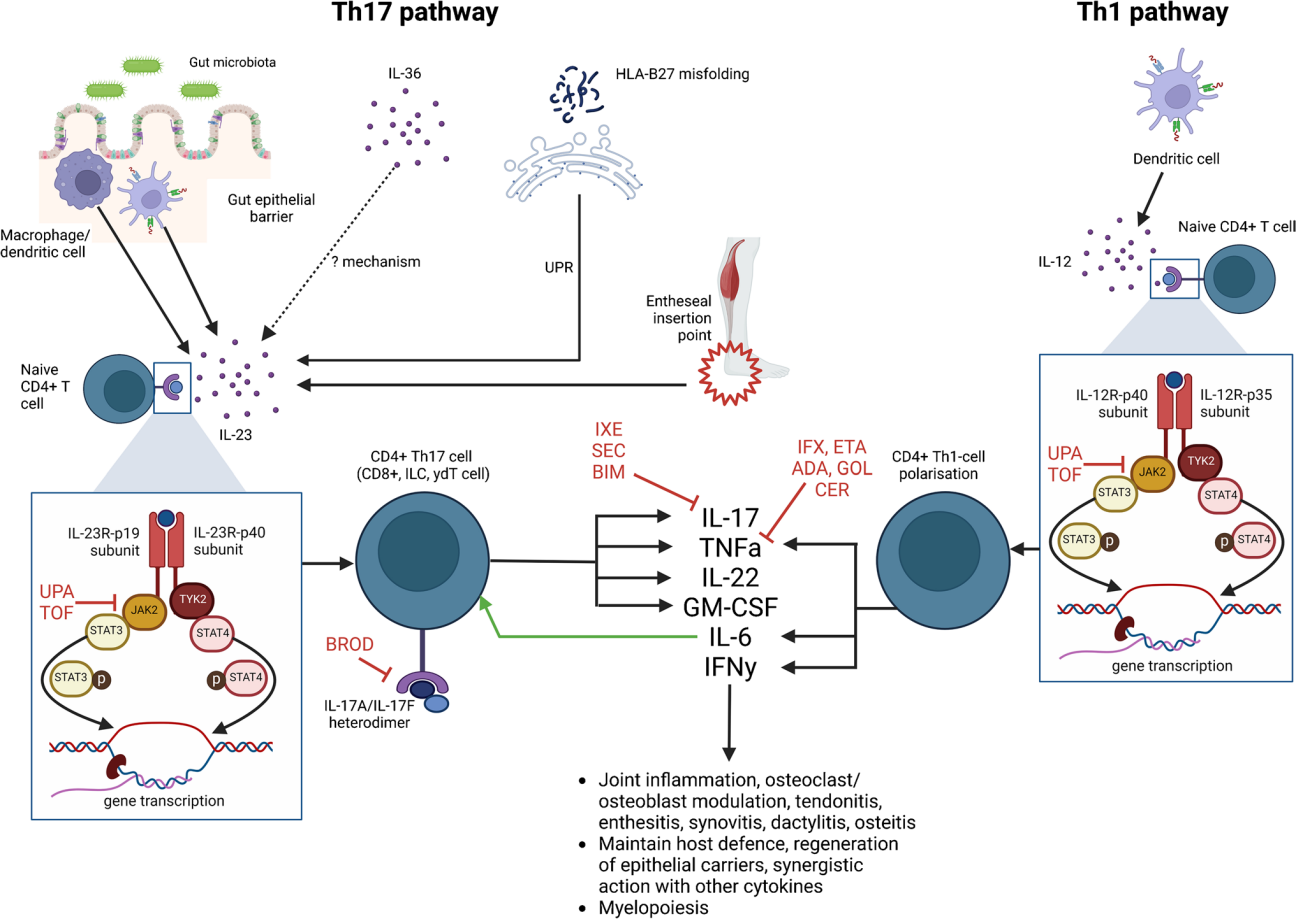
The two major inflammatory cell types thought to be implicated in axSpA are Th1 and Th17 CD4 + T cells. Naïve T cells are polarised to Th17 cells in the presence of IL-23, IL-6 and TGFβ. IL-23 may be activated in a number of ways including gut microbiota-host dendritic cell interactions, activation of tissue-resident cells due to entheseal stress, unfolded protein response (UPR) in triggered by misfolded HLA-B27, or via IL-36 though the mechanisms of the latter are poorly understood. IL-23R activation triggers downstream JAK/STAT pathway signalling and gene transcription, polarising naïve CD4 + T cells to Th17-cells. Th17 cells produce cytokines including IL-17 and IL-22 which drive inflammation. Th1 polarisation is driven by IL-12 which also signals via the JAK/STAT pathway. Th1 cells produce TNFα, IL-6 and IFNγ which promote inflammatory pathways involved in axSpA. As discussed above, IL-6 is also required for Th17 cell polarisation. All bDMARDs used in axSpA target one or more of these inflammatory pathways, resulting in direct and indirect inhibition of inflammatory pathways associated with axSpA. Key: ADA, adalimumab; BIM, bimekizumab; BROD, brodalumab; CER, certolizumab; ETA, etanercept; GM-CSF, granulocyte–macrophage colony-stimulating factor; GOL, golimumab; IFNy, interferon-gamma; ILC, innate lymphoid cells; IFX, infliximab; IL, interleukin; IXE, ixekizumab; JAK, janus kinase; p, phosphorylation; SEC, secukinumab; STAT, signal transducers and activators of transcription; TNFα, tumour necrosis factor- alpha; TOF, tofacitinib; UPA, upadacitinib; UPR, unfolded protein response; γδT, gamma-delta T cells

Detailed studies of the molecular biology of axSpA came a little later leading to large Phase III trials with agents such as IL-17is, IL-23is and JAKis, opening up new and exciting drug development opportunities specific to this disease [34]. However, it is important to note that the majority of drug development is based on studies of established r-axSpA/AS, derived from pre-clinical models or from studies in other IMIDs; with more work needed to explore differences between these and real-world clinical models [35]. Finally, cellular immunity (the target for all bDMARDs) is only a small piece of the puzzle, since the pathogenesis of axSpA is also shaped by a number of other mechanical, environmental and genetic factors that could not possibly be covered in this focused review on therapeutics [36–38]. Figure 1 provides an overview of the current understanding of the molecular mechanisms of axSpA in relation to existing bDMARDs. The rest of this review details exactly how our understanding of molecular mechanisms in axSpA has been shaped by bDMARDs.

## TNF Inhibitors

TNFα is a pleiotropic cytokine with a plethora of direct and indirect effects on both innate and adaptive immunity [39]. The main cellular sources of TNFα in axSpA are monocytes/macrophages, but it may also be produced by natural killer (NK) cells, T cells, neutrophils and tissue-resident non-immune cells such as fibroblasts [40, 41]. In axSpA, TNFα contributes to pathology primarily via modulation of innate immune responses and, to a lesser extent, Th1 and Th17 signalling (Fig. 1) [42–45]. The net effect of these actions is increased production of IL-1, IL-6 and other pro-inflammatory mediators, recruitment of adaptive immune cells (T and B cells) and macrophages, Th1 polarisation of CD4 + T cells, tissue inflammation and ultimately propagation of the immune response [44]. In addition, the majority of axSpA patients are positive for the HLA-B27 allele (up to 90% in some studies) [46, 47]. In vitro, axSpA patients have high levels of NK and CD4 + T cells expressing KIR3DL2 which is capable of recognising HLA-B27 homodimers expressed on the surface of cells triggering activation and release of IFNy, TNFα and IL-17 [48, 49]. IL-17 works synergistically with TNFα to promote the release of downstream inflammatory mediators and modulate bone metabolism [44, 50], as well as contributing to the pathogenesis of axSpA via other TNF-independent mechanisms (discussed later in this review).

Given the above, it is unsurprising that TNFis are effective for axSpA. However, the response is not ubiquitous, and over a third of patients experience non-response (NR) to their first TNFi [51, 52]. Possible explanations include non-compliance with treatment, individual differences in drug metabolism, pharmacodynamics and pharmacokinetics or the development of anti-drug antibodies [53, 54]. Several studies from a decade ago linked HLA-B27 misfolding with downstream TNFα production [55]; an observation that was then confirmed in a recent meta-analysis showing HLA-B27 positivity was associated with improved BASDAI50 response to TNFis compared with HLA-B27 negative disease [56]. Furthermore, a smaller study showed a significantly improved response in HLA-B27 allele homozygous individuals compared with heterozygous [57]. On the other hand, there have been no clinically significant differences reported for bDMARD response rates between r-axSpA and nr-axSpA for any of the different biologic agents to date (Table 1) [58].

**Table 1:** Summary of licensed bDMARDs in axSpA and their efficacy on different disease features

bDMARD	Year licensed by NICE	License (r- and nr- axSpA)	Efficacy				
			Axial disease	Peripheral disease	Skin psoriasis	Uveitis	IBD
Etanercept	2003	r-	x	x	x		x
Infliximab	2004	r- and nr-	x	x	x	x	x
Adalimumab	2008	r- and nr-	x	x	x	x	x
Golimumab	2009	r-	x	x	x	x	x
Certolizumab	2013	r- and nr-	x	x	x	x	x
Secukinumab	2016	r- and nr-	x	x	x		
Ixekizumab	2021	r- and nr-	x	x	x		x**
Upadacitinib		r- and nr-	x	x	x		x**
Tofacitinib		r- and nr-	x	x	x		
Bimekizumab	2022*	r- and nr-					

*Key*: *axSpA *axial spondylarthritis, *bDMARD *biological disease-modifying anti-rheumatic drug, *nr *non-radiographic, *r *radiographic

^*^Pre-authorisation granted full document to be published Aug 2022

^**^For treatment of ulcerative colitis only

Following TNFi NR, the National Institute of Clinical Excellence (NICE) allows the use of a second TNFi or an IL-17i in axSpA [8]. Whilst some axSpA patients will respond to a second TNFi, overall response rates to a second bDMARD (TNFi or IL-17i) are lower [59], pointing to a possible change in an individual’s immune environment, and those rates of NR may be worse for those with primary NR compared with secondary NR. Manica et al. showed no difference in Ankylosing Spondylitis Disease Activity Score Clinically Important Improvement (ASDAS-CII) for the second TNFi between those with primary NR versus secondary NR to their first TNFi; however, there was a difference when the more stringent ASDAS inactive disease (ASDAS-ID) outcome measure was used [60]. The underlying molecular basis for bDMARD NR in axSpA remains poorly characterised.

Another indication supporting that an individual’s immunobiology can change with treatment, and/or with the duration of disease, is the observation of NR after re-challenge with the same bDMARD following an interruption to treatment, e.g. for surgery or whilst receiving treatment for a concurrent infection [61]. Infections themselves may prime the immune system and can be a trigger for new autoimmune disease or change in the manifestations of an existing one; the classic example being reactive arthritis [62]. The infectious challenge to the immune system can result in loss of efficacy even in patients with years of good response.

Given the above, it was suggested that combining bDMARD therapies might be more efficacious in some patients; a theory tested by Hammoura et al. who used a murine model to test whether dual inhibition of IL-17 and TNFi would be superior to TNFi and/or IL-17i. Unfortunately, efficacy was similar across all 3 treatment arms suggesting no additive or synergistic effects of combining different classes of bDMARD treatment [63]. However, all mice were treatment-naïve at the time of study entry, and so the efficacy of dual-blockade in the setting of primary or secondary NR to one or more bDMARDs remains unexplored. Similar results were found in humans where dual inhibition of TNFα and IL-17A with ABT-122 was shown to have a similar efficacy and safety profile to that of the TNFi alone in trials of rheumatoid arthritis and psoriatic arthritis [64] with limited real-life reports of highlighted an enhanced side effect risk profile in resistant SpA [65].

TNFis are not universally efficacious across all EMMs, and in some cases may even exacerbate them. Notably, etanercept has been linked with an increased risk of uveitis [66] as well as other ocular complications including intermediate uveitis, posterior uveitis, scleritis and very rarely orbital myositis [67]. Current recommendations are to avoid etanercept in patients who have or who go on to develop uveitis [66]. Yet paradoxically, other TNFis are the preferred choice of bDMARD for uveitis [68]. The explanation for this differential effect could lie in the unique mode of action of etanercept when compared with other TNFis. Etanercept, a soluble receptor blocker, blocks the receptor but does not remove circulating TNFα like the other TNFi agents on the market [68] and there are some data to suggest its larger molecular weight results in poorer intraocular permeability, reducing efficacy [68, 69]. Indeed, two RCTs failed to show the superiority of etanercept over placebo in the treatment of ocular pathologies [70, 71]. However, this would not account for the paradoxically increased incidence of uveitis that has also been observed in some studies [66]. Perhaps one reason for the difference is that etanercept also blocks TNFβ, however in murine models of uveitis TNFβ levels are also increased, therefore one would expect this to help treat uveitis and not cause it [72]. Another study showed that TNF receptor (TNF-R) and TNFα levels were elevated in ocular fluids from patients with active uveitis, and blocking the TNF-R resulted in an increase in TNFα production by T cell populations. The authors therefore postulate that the TNF-R might have a regulatory role in uveitis over and above just mopping up TNFα [73]. Clearly further research is needed to fully understand the reasons for etanercept failure in the treatment of uveitis [74].

## IL-17 Inhibitors

IL-17 plays an important role in the defence against fungal and certain bacterial pathogens. It was first linked to human disease through a model of mouse autoimmune encephalitis in 2005 [75], heralding the discovery of IL-17 pathway dysregulation in several autoimmune and autoinflammatory diseases including axSpA [76]. The IL-17 family includes 6 known cytokines (IL-17A-IL17F) [77]. The most important in axSpA are IL-17A and IL-17F which form heterodimers capable of activating the IL-17RA and IL-17RC complex on target cells [78]. Several cells are capable of IL-17 including CD4 + T cells, CD8 + T cells, mucosal-associated invariant T cells (MAIT cells), innate lymphoid cells (ILCs), gamma delta T cells (γδT-cells) and invariant natural killer T cells (iNKTs), neutrophils, mast cells and eosinophils [78]. Although a number of these cells may play a role in IL-17 production in axSpA, most IL-17 is thought to be produced by Th17 cells [79–81]. IL-17 production, in turn, stimulated IL-1β, TNFα, IL-6 and IL-23 by synovial fibroblasts, monocytes and macrophages generating a positive feedback loop for further Th17 cell differentiation [82]. In addition to IL-17, Th17 polarised cells produce other cytokines (GM-CSF), chemokines (CXCL1, XCL2, CXCL8, CCL20, etc.), anti-microbial peptidases, matrix metalloproteinases, complement and other acute phase reactants [83, 84]. This pro-inflammatory storm ultimately results in dysregulated bone metabolism, axial and peripheral joint inflammation and enthesitis in both mouse models and in-vitro studies of peripheral blood and tissues from patients with axSpA [78, 85].

Presently, two IL-17A inhibitors, secukinumab and ixekizumab, have been licensed by NICE, the Medicines and Healthcare products Regulatory Agency (MHRA) and Food and Drug Administration (FDA) for use in axSpA either as 1st or 2nd line treatment [9, 10, 86–89]. Bimekizumab (an IL-17A and IL-17F inhibitor) has also now received pre-authorisation [16] with results from Phase III trials showing similar efficacy to TNFi (Table 1) [90]. Although to date, there are no head-to-head comparator studies in axSpA, adalimumab was used as a comparator in the COAST-V trial, and ixekizumab demonstrated a greater absolute improvement in ASAS-40 scores between week 0 and week 16 compared to adalimumab, although the study was not powered to detect significance [91]. Despite largely similar efficacy on disease control overall, there are some important differences with regards to EMMs. IL-17 inhibition showed superior efficacy on skin psoriasis compared with TNFis, and therefore axSpA patients with difficult-to-treat skin psoriasis may warrant earlier used IL-17is as opposed to a TNFis [92]. Similarly, psoriatic arthritis with a predominantly axial pattern of joint involvement may benefit from the early introduction of IL-17i because conventional non-biological DMARDs, although effective for skin psoriasis and peripheral joint inflammation, may not effectively treat axial disease [93–95].

Changes of axSpA are present in 10–36% of IBD patients, therefore IBD is of major clinical significance in axSpA [96]. TNFα is are effective treatment for IBD [97]. However, trials have demonstrated an increased incidence of IBD, as well as exacerbation of IBD, in axSpA patients treated with IL-17i [98]. The same observations were made in studies of IL-17 is in non-axSpA IBD, with two clinical trials of the IL-17 is secukinumab and brodalumab stopped early due to worsening of IBD symptoms in Crohn’s disease patients [99, 100]. These results perplexed experts initially as earlier studies pre-clinical and genetic studies had demonstrated dysregulation of the IL-17/23 axis in IBD [98]. However, studies have since also shown that IL-17 inhibition may interfere with dysregulation of the gut epithelial barrier, predisposing to infection and inflammation which can, in turn, exacerbate or trigger IBD [98]. Additionally, in a murine model of autoimmune uveitis, authors demonstrated IL-17A may be important for negative feedback of pro-inflammatory Th17 cell responses. They showed that IL-17A, through NFκβ, induces IL-24 production by Th17 cells, thereby downregulating IL-17F and GM-CSF to suppress Th17 cell activity [101]. On the other hand, there is evidence that the effects of IL-24 are dose-dependent, with low doses suppressing Th1 cells and higher doses promoting Th1 and Th17 cell activity in the study of colorectal cancer [102]. IL-24 also acts on the IL-20 receptors on epithelial cells in the colonic mucosa where it plays a regulatory role [103] and high levels of IL-24 have also been observed in patients with skin psoriasis and in RA synovial fluid [103]. Altogether, these studies suggest a differential role for IL-17A, IL-17F and their downstream cytokines in the inflammatory process which may be cell- and tissue-specific, and may account for the lack of efficacy of IL-17is in IBD. It also suggests that dual IL-17A/IL-17F inhibition may have other effects, and therefore that agents like bimekizumab may behave differently to agents that block IL-17A only, however more work is needed in this area [104].

## IL-23 Inhibitors

IL-23 is another cytokine which works in conjunction with IL-17 in the pathogenesis of axSpA [105]. Structurally, IL-23 is a heterodimer complex of two subunits, p40 and p19, which when combined interact with the IL-23R on target cells triggering downstream activation of JAK/STAT signalling pathways and transcription of several pro-inflammatory mediators including IL-17, IL-22 and TNFα [106, 107]. Several animal studies demonstrate the importance of IL-23 in the pathogenesis of SpA [108–113]. Moreover, IL-23R and STAT2/3 risk alleles were reported amongst the non-HLA-B27 associations for axSpA in a recent meta-analysis of ankylosing spondylitis genome-wide association studies [114, 115]. Although individual risk effect estimates were small, this is true for all non-HLA-B27 risk alleles and does not necessarily mean they are not relevant to disease in at least some patients, particularly given that functional studies of the role of IL-23 in axSpA suggest otherwise. IL-23 and IL-17 levels are elevated in peripheral blood from AS patients [116–118], and IL-23 can stimulate IL-17 production by a number of cells including CD8 + T cells, γδT-cells as well as other lymphoid cell lines [83–85].

Given these findings, and the success of four IL-23 inhibitors (the p19IL-23 inhibitor risankizumab, the p40IL-12/23 inhibitor ustekinumab, the p19IL-23 inhibitor IL-23 inhibitor guselkumab and the p19IL-23 inhibitor tildrakizumab) in treating patients with skin psoriasis and/or PsA [119–129], many thought IL-23 inhibitors would be a viable treatment option in axSpA. Yet despite early promising results from a German open-label study of IL-23 inhibitors, Phase III trials failed to demonstrate efficacy [21–24]. These trials used comparable patient groups and similar endpoints to other bDMARD trials in axSpA, and therefore, lack of efficacy cannot be accounted for simply on the basis of trial design [105].

To understand the possible cause for the lack of efficacy of IL-23 inhibitors in axSpA requires a deeper understanding of the IL-17/IL-23 signalling pathway and differences that might exist in different tissue compartments and at different time points in the disease trajectory [130]. Recent studies on the spinal human enthesis have shown evidence of IL-17A production independent of IL-23R expression [131]. Furthermore, there are myeloid cells resident in the entheses of the spine capable of producing IL-23 in response to mechanical stress factors [132]. Other potential sources of IL-23 in axSpA that have been proposed include, cellular endoplasmic reticulum stress responses in relation to misfolded HLA-B27 proteins, aberrant host response to a microbial insult (as observed in reactive arthritis patients) and IL-36 production, although there is very little research on the latter [133]. Yet robust evidence that any of these pathways are clinically important and are active in-vivo, is yet to be obtained. Moreover, whilst these theories offer an explanation to the original source of IL-23 production, in established disease, data suggest that these may no longer be important, since Th17 cells can produce IL-17 independent of IL-23 as discussed above. Furthermore, it raises the question of whether IL-23 inhibition may only be of value in the very early or pre-clinical disease state, which has yet to be defined in axSpA [134].

Alternatively, it is possible that there is a subset of patients who continue to show a IL-23-driven disease who might benefit from IL-23is, or that certain EMMs are more driven by IL-23 than others owing to tissue-specific factors. Of even more significant interest is perhaps the fact that IL-23i, somewhat counterintuitively may be efficacious in axial psoriatic arthritis. Post hoc analysis of the axial PsA patient subsets from larger PsA trials of IL-23 inhibitors (PSUMMIT, DISCOVER1 and DISCOVER2) showed patients still felt joint symptoms had improved [105, 120, 124, 125]. The caveat to this is that all primary outcome measures used in the trials measured disease activity as a whole, which includes peripheral disease, skin psoriasis and axial disease. Furthermore, no MRI imaging was available to correlate symptomatic improvement with objective evidence of a reduction in spine/SIJ inflammation on spinal MRI. Nevertheless, this observation is of great interest and there is a need for dedicated trials of IL-23i in axial PsA focusing on their impact on axial disease specifically.

## JAK Inhibitors

Cytokines signal via numerous downstream pathways, one of which is the Janus Kinase/ signal transducers and activators of transcription (JAK/STAT) [135]. The JAK/STAT family includes JAK 1,2,3 and tyrosine kinase 2 (TYK2), which is associated with the intracellular aspect of type I/II cytokine receptors on the cell surface, including the IL-23R, IL-6R, type 1 and type 2 IFN receptors, IL-7R and GM-CSF receptors. When activated, JAKs phosphorylate themselves and their receptors, then dephosphorylate after 15–30 min to prevent permanent receptor activation. STAT molecules bind during the active state and are phosphorylated, whereupon they migrate to the nucleus and bind to target genes promoting transcription. JAKis in commercial use have variable selectivity for particular JAK family members, allowing for some degree of differential receptor modulation [136]. The two JAKis currently licensed for the treatment of axSpA; upadacitinib and tofacitinib; selectively target JAK1, but also may interact with other JAKs, therefore have a broad spectrum of activity on the receptor subtypes above. The downstream effect is the modulation of several innate and adaptive immune processes contributing to axSpA including Th1 and Th17 differentiation, growth/maturation of lymphoid cells, and tissue inflammation. A detailed review of the specific downstream effects of JAKis can be found here [137].

JAKis demonstrated clear efficacy for the treatment of axSpA in trials meeting their primary and secondary endpoints with similar outcomes as reported in trials of TNFi and IL-17is [138–140]. However, as JAKis have only just been authorised for the treatment of axSpA, more data are needed to establish their efficacy against EMM such as IBD and AU (Table 1) and to fully understand their mechanism(s) of effect in axSpA. JAKis target several inflammatory pathways, but not all are directly involved in axSpA, therefore the mechanism of their effect on disease is likely indirect. With this comes the risk of further unwanted and/or unanticipated side effects. Indeed, this has already been observed in RA, with emerging real-world clinical data demonstrating an increased risk of cardiovascular and thrombotic events in the RA population. Accordingly, JAKis should not be used in those at high risk for thrombosis or those over the age of 65 [141, 142]. On the other hand, JAKis have been in use in RA for a number of years with no apparent increase in incidence of IBD, psoriasis or uveitis [143]. Furthermore, tofacitinib and upadacitinib are actually approved by the FDA, MHRA and NICE for the treatment of ulcerative colitis [141, 142, 144–147]. Upadacitinib but not tofacitinib, has shown favourable results in Crohn’s disease, although regulatory approval for use in Crohn’s disease has yet to be granted [148, 149]. Nevertheless, these studies suggest that, like TNFis, JAKi might have beneficial effects on both the joints and gut of patients with axSpA and IBD [150]. JAKis have been trialled in psoriatic arthritis, but not skin psoriasis specifically. Despite this, trial data suggested that the improvement in the psoriatic arthritis severity index (PASI) reflected the beneficial effect of JAKis on both arthritis and skin psoriasis [136].

## Potential Future bDMARDs

As described previously, new bDMARDs are in late-phase development, including a novel IL-17R blocker brodalumab, with a different mode of action of IL-17 direct cytokine inhibition [14]. Similarly, other JAKi may soon be licensed, and it will be interesting to see if/how their different receptor selectivity impact on the efficacy of treatment. Finally, there is also a new class of agent in early trial phases, nanilumab, a GM-CSF inhibitor (19). The role of GM-CSF was first suggested as working upstream of the IL-17/23 inhibitor axis in triggering the disease, and early-phase trials have shown some success in axSpA and RA [151]. If trials are successful, this could one day become another class of bDMARD for axSpA.

## Summary

There are now a significant array of biologic and targeted synthetic drugs available for the treatment of axSpA, each with somewhat divergent effects on axial/peripheral joint symptoms and EMMs. TNFis show efficacy for the treatment of axial/peripheral disease, enthesitis, IBD and uveitis (except for etanercept). IL-17 inhibitors treat axial/peripheral joint symptoms and are particularly useful in the presence of concomitant skin psoriasis. On the other hand, they may trigger or exacerbate flares of IBD and are second to TNFis for the treatment of uveitis. JAKis have established efficacy on peripheral and axial symptoms in trials but effects on EMMs are as yet unknown. How and why these differences might exist between drugs, and how this divergence relates to the underlying molecular pathogenesis, remains unclear. Specific studies in pre-clinical and early disease may now be possible with increased awareness of the disease, advancing imaging methods and the growing use of predictive genomics technologies across the whole spectrum of IMIDs, and may reveal new drug targets that can tell us more about the disease in its early stages. At the same time, results of ongoing pharmacovigilance studies of existing drugs over the coming years may reveal new insights into how these treatments affect the pathogenesis of axSpA in the short- and long-term, which may further change our understanding of the biology of this disease throughout its course. Even more valuable will be the data on how they impact particular subsets of patients (nr- vs r-, HLA-B27 allele positive versus negative, males vs. females, etc.) which may account for some differences in the observed treatment efficacy. Twenty years of biologics for axSpA have already taught us a lot about this disease. With well-planned drug development and post-market surveillance, the next 20 years are likely to teach us even more.

## Data Availability

Not applicable
