# Correction to: Have Therapeutics Enhanced Our Knowledge of Axial Spondyloarthritis?

**DOI:** 10.1007/s11926-023-01101-0

**Published:** 2023-04-03

**Authors:** S. R. Harrison, H. Marzo-Ortega

**Affiliations:** 1 The University of Leeds, Leeds Institute for Rheumatic and Musculoskeletal Medicine (LIRMM), NIHR Leeds Biomedical Research Centre, Leeds Teaching Hospitals Trust, Leeds, UK; 2 The University of Leeds, Leeds Institute of Cardiovascular and Metabolic Medicine, the LIGHT building, Clarendon Way, Leeds, UK

**Correction to: Current Rheumatology Reports (2023) 25:56-67**

10.1007/s11926-023-01097-7

In this article, the section heading was incorrectly given as ‘IL-17 Inhibitorsfailure in the treatment of‘ but should have been ‘IL**-**17 Inhibitors‘.

The original article has been corrected.

